# Ginkgolide B and bilobalide block the pore of the 5-HT_3_ receptor at a location that overlaps the picrotoxin binding site

**DOI:** 10.1016/j.neuropharm.2010.11.003

**Published:** 2011-02

**Authors:** Andrew J. Thompson, Gavin E. Jarvis, Rujee K. Duke, Graham A.R. Johnston, Sarah C.R. Lummis

**Affiliations:** a Department of Biochemistry, University of Cambridge, Tennis Court Road, Cambridge CB2 1QW, UK; b Department of Pharmacology, University of Sydney, Sydney NSW 2006, Australia

**Keywords:** Serotonin, Cys-loop, Picrotoxin, Antagonist, Channel, nACh, nicotinic acetylcholine, GABA, γ-aminobutyric acid, 5-HT, 5-hydroxytryptamine/serotonin, GA, ginkgolide A, GB, ginkgolide B, BB, bilobalide, PTX, picrotoxin, PXN, picrotoxinin, PTN, picrotin

## Abstract

Extracts from the *Ginkgo biloba* tree are widely used as herbal medicines, and include bilobalide (BB) and ginkgolides A and B (GA and GB). Here we examine their effects on human 5-HT_3_A and 5-HT_3_AB receptors, and compare these to the effects of the structurally related compounds picrotin (PTN) and picrotoxinin (PXN), the two components of picrotoxin (PTX), a known channel blocker of 5-HT_3_, nACh and GABA_A_ receptors. The compounds inhibited 5-HT-induced responses of 5-HT_3_ receptors expressed in *Xenopus* oocytes, with *IC*_50_ values of 470 μM (BB), 730 μM (GB), 470 μM (PTN), 11 μM (PXN) and >1 mM (GA) in 5-HT_3_A receptors, and 3.1 mM (BB), 3.9 mM (GB), 2.7 mM (PTN), 62 μM (PXN) and >1 mM (GA) in 5-HT_3_AB receptors. Radioligand binding on receptors expressed in HEK 293 cells showed none of the compounds displaced the specific 5-HT_3_ receptor antagonist [^3^H]granisetron, confirming that they do not act at the agonist binding site. Inhibition by GB at 5-HT_3_A receptors is weakly use-dependent, and recovery is activity dependent, indicating channel block. To further probe their site of action at 5-HT_3_A receptors, BB and GB were applied alone or in combination with PXN, and the results fitted to a mathematical model; the data revealed partially overlapping sites of action. We conclude that BB and GB block the channel of the 5-HT_3_A receptor. Thus these compounds have comparable, although less potent, behaviour than at some other Cys-loop receptors, demonstrating their actions are conserved across the family.

## Research highlights

► Ginkgolide B and bilobalide inhibit 5-HT3 receptors. ► They are more potent at 5-HT_3_A than 5-HT_3_AB receptors. ► BB and GB block the open channel of 5-HT_3_A receptors. ► Their sites of action partially overlap with those of the channel blocker picrotoxinin.

## 1 Introduction

**Fig. 1:**
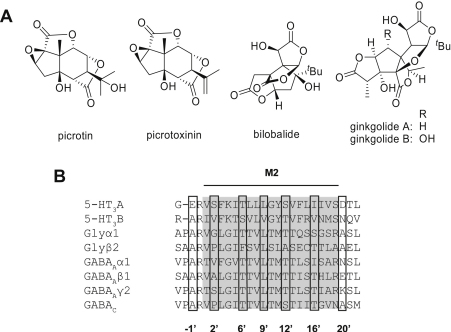
Structures of the compounds used in this study (Panel A), and an alignment of human Cys-loop receptor M2 regions (Panel B). The alignment includes the human 5-HT_3_ receptor A and B subunits in addition to subunits from other Cys-loop receptors that are inhibited by BB, GB and PTX. M2 residues are usually referred to using prime (′) notation, which is shown below the alignment. Accession numbers for the alignment are: 5-HT3A (P46098), 5-HT3B (O95264), glycine α1 (P23415), glycine β2 (P48167), GABA_A_ α1 (P14867), GABA_A_ β1 (P18505), GABA_A_ γ2 (Q8N1C3).

Flavonoids and terpene lactones are the two major types of active ingredients extracted from the leaves, roots and bark of the *Ginkgo biloba* tree (Chen et al., 2007; van Beek, 2005). The clinically relevant leaf extract EGb761 is enriched in these compounds and contains, among others, the terpene lactones bilobalide (BB) and ginkolides A and B (GA, GB). Picrotoxin (PTX), a GABA receptor antagonist, is also plant derived (from the fruit of *Anamirta cocculus*), and contains two components that are structurally related to these *G. biloba* constituents (Fig. 1A; picrotoxinin (PXN) and picrotin (PTN)). Like PTX, BB and the ginkgolides are inhibitors of ionotropic GABA and glycine receptors, and all of these compounds bind in the receptor channel (Fig. 1B; Heads et al., 2008; Erkkila et al., 2008; Huang et al., 2004; Hawthorne et al., 2006; Kondratskaya et al., 2005).

GABA and glycine receptors, like 5-HT_3_ and nACh receptors, are members of the Cys-loop family of neurotransmitter-gated receptors. These proteins play a major role in fast synaptic transmission in both the central and peripheral nervous systems. They consist of five symmetrically arranged subunits, each of which contains a large extracellular N-terminal domain, four transmembrane α-helices (M1–M4), of which M2 lines the central ion-conducting pore, and a large intracellular loop between M3 and M4 which is responsible for channel conductance and intracellular modulation (Thompson and Lummis, 2006a,b). GABA and glycine receptors are chloride-selective, and activation generally results in a hyperpolarising current that is inhibitory. Classic inhibitors of these receptors such as bicuculline, picrotoxin and strychnine cause uncontrolled muscle contractions and/or convulsions (Jarboe et al., 1968). As the ginkgolides are also antagonists of these receptors, it is surprising that they are not also convulsants, but instead may have anti-convulsant, neuro-protective, anxiolytic and other beneficial properties (Ahlemeyer and Krieglstein, 2003; Kiewert et al., 2007; Sasaki et al., 1997). Given the sequence similarity of channel-lining regions within the Cys-loop family, and the fact that PTX blocks 5-HT_3_ receptor responses, it is possible that the ginkgolides also bind to the 5-HT_3_ receptor, which might provide some explanation for these different properties.

In this study we compare the inhibitory effects of BB, GA, GB, PTN and PXN on 5-HT_3_A and 5-HT_3_AB receptors. Using two-electrode voltage-clamp of *Xenopus* oocytes and competition radioligand binding on transfected HEK 293 cells we provide the first account of BB and GB inhibition of 5-HT_3_ receptors, and probe their mechanisms of action at the 5-HT_3_A receptor.

## 2 Materials and methods

### 2.1 Materials

PXN and PTN were separated and purified by recrystallisation following short column vacuum chromatography from PTX purchased from Sigma–Aldrich Pty. Ltd (NSW, Australia). BB, GA and GB were isolated from a 50:1 *G. biloba* leaf extract purchased from Winshing Pty Ltd (NSW, Australia) and purified by short column chromatography and recrystallisation. The ^1^H and ^13^C NMR spectra of the purified compounds were consistent with the published data (Perry et al., 2001; van Beek, 2005), and indicated purity > 98% in all cases. Human 5-HT_3_A (accession number: P46098) and 5-HT_3_B (O95264, both kindly donated by John Peters), and mouse 5-HT_3_A (Q6J1J7) and 5-HT_3_B (Q9JHJ5, kindly donated by Ewan Kirkness) were used.

### 2.2 Receptor expression

Human 5-HT_3_A and 5-HT_3_B subunit cDNAs were cloned into pGEMHE for oocyte expression (Liman et al., 1992) and pcDNA3.1 (Invitrogen, Paisley, U.K.) for expression in HEK 293 cells. cRNA was *in vitro* transcribed from linearised pGEMHE cDNA template using the mMessage mMachine T7 Transcription kit (Ambion, Austin, Texas, USA). Stage V and VI oocytes were injected with 50 nl of ∼300 ng μl^−1^ cRNA, and currents were recorded 1–4 days post-injection. A ratio of 1:3 (A:B) was used for the expression of heteromeric 5-HT_3_ receptors.

### 2.3 Oocyte maintenance

*Xenopus laevis* oocyte positive females were purchased from NASCO (Fort Atkinson, Wisconsin, USA) and maintained according to standard methods. Harvested stage V-VI *Xenopus* oocytes were washed in four changes of ND96 (96 mM NaCl, 2 mM KCl, 1 mM MgCl_2_, 5 mM HEPES, pH 7.5), de-folliculated in 1.5 mg ml^−1^ collagenase Type 1A for approximately 2 h, washed again in four changes of ND96 and oocytes stored in ND96 containing 2.5 mM sodium pyruvate, 50 mM gentamycin, 0.7 mM theophylline.

### 2.4 Cell culture and transfection

Human embryonic kidney (HEK) 293 cells were maintained on 90 mm tissue culture plates at 37 °C and 7% CO_2_ in a humidified atmosphere. They were cultured in DMEM:F12 with GlutaMAX™ I media (Dulbecco’s Modified Eagle’s Medium /Nutrient Mix F12 (1:1), Invitrogen, Paisley, UK) containing 10% foetal calf serum. For radioligand binding studies, cells in 90 mm dishes were transfected using polyethyleneimine (PEI): 30 μl PEI (1 mg /ml), 5 μl cDNA and 1 ml DMEM were incubated for 10 min at room temperature, added drop wise to a 80–90% confluent plate, and incubated for 3–4 days before use.

### 2.5 Electrophysiology

Using two electrode voltage clamp, *Xenopus* oocytes were clamped at −60 mV using an OC-725 amplifier (Warner Instruments, Connecticut, USA), Digidata 1322A and the Strathclyde Electrophysiology Software Package (Department of Physiology and Pharmacology, University of Strathclyde, UK). Currents were filtered at a frequency of 1 kHz. Micro-electrodes were fabricated from borosilicate glass (GC120TF-10, Harvard Apparatus, Edenbridge, Kent, UK) using a two stage horizontal pull (P-87, Sutter Instrument Company, California, USA) and filled with 3 M KCl. Pipette resistances ranged from 0.5–1.5 MΩ. Oocytes were perfused with saline at a rate of 15 ml min^−1^. Drug application was via a simple gravity fed system calibrated to run at the same rate. Extracellular saline contained (mM), 96 NaCl, 2 KCl, 1 MgCl_2_ and 5 mM HEPES; adjusted to pH 7.4 with NaOH.

Analysis and curve fitting was performed using Prism V3.02 (GraphPad Software, San Diego, California, USA, www.graphpad.com). Concentration-response data for each oocyte was normalised to the maximum current for that oocyte. For inhibition curves, antagonists were routinely co-applied in the presence of agonist. For PXN, oocytes were also pre-treated with the compound for 20 s. A 2 min wash was used between drug applications. The mean and S.E.M. for a series of oocytes was plotted against agonist or antagonist concentration and iteratively fitted (GraphPad Prism v3.02) to the following Hill equation:

(1) $$I_{A}=I_{\min}+\frac{I_{\max}-I_{\min}}{1+10^{n_{\text{H}}\left(\log A_{50}-\log\;A\right)}}$$

where *A* is the concentration of ligand present; *I_A_* is the current in the presence of ligand concentration *A*; *I*_min_ is the current when *A* = 0; *I*_max_ is the current when *A* = **∞** , *A*_50_ is the concentration of *A* which evokes a current equal to (*I*_max_ + *I*_min_)/2; and *n*_H_ is the Hill coefficient. Results are presented as mean ± S.E.M.. Statistical analysis was performed using ANOVA in conjunction with a Dunnett’s post-hoc test.

In order to evaluate whether different channel blocking compounds shared a binding site (scenario 1) or bound at discrete sites (scenario 2) the effect of co-application of the compounds was compared with a predicted outcome for each scenario given the observed effects of the two compounds individually using equations from Kenakin (1997) and analysed using SPSS16.0 (SPSS Inc., Ilinoi, USA). 97)

*Scenario 1*: Where compounds share a binding site it was assumed that the extent of binding would be determined by a competitive interaction between the compounds such that the proportion of binding sites occupied (*P*_occ_) in the presence of two competing compounds is given by the following expression:

(2) $$P_{\text{occ}}=\frac{AK_{A}+BK_{B}}{1+AK_{A}+BK_{B}}$$

where *A* and *B* are the concentrations of two ligands and *K_A_* and *K_B_* are their affinity constants respectively.

If it is assumed that receptor occupancy is directly proportional to receptor blockade, it follows that where *A* alone causes a functional inhibition of *I_A_* (and likewise for *B*), then the inhibition observed in the presence of *A* and *B (I_AB_*_1_*)*, where the two compounds compete for the same site, is given by the following expression:

(3) $$I_{AB1}=\frac{I_{A}+I_{B}-\left(2I_{A}I_{B}\right)}{1-\left(I_{A}I_{B}\right)}$$

*Scenario 2*: Where the two compounds bind in discrete locations and therefore do not interfere with each other’s binding, the proportion of receptors occupied by one or both compound, and therefore the inhibition observed in the presence of *A* and *B* (*I_AB_*_2_), will be given by the following expression:

(4) $$I_{AB2}=I_{A}+I_{B}-I_{A}I_{B}$$

In each individual experiment, for each combination of inhibitors, data values were obtained for *I_A_*, *I_B_* and *I_AB_*. From *I_A_* and *I_B_* predicted values of *I_AB_*_1_ and *I_AB_*_2_ were also derived. A two-way ANOVA was performed on values of *I_AB_*, *I_AB_*_1_ and *I_AB_*_2_ in which the distinction between *I_AB_*, *I_AB_*_1_ and *I_AB_*_2_ was treated as a fixed factor and the variation in levels of inhibition between experiments was treated as a random factor. Where the ANOVA indicated a significant difference (*P* < 0.05) between *I_AB_*, *I_AB_*_1_ and *I_AB_*_2_ a post-hoc analysis was carried out using the Waller–Duncan method to identify homogenous subsets with a significance level of 0.05 and a Type I /Type II error ratio if 100.

Our models were based on the following assumptions: 1. that the channel blocking compounds only effect the response that is seen when the channel is open. Hence, in the absence of the agonist 5-HT, the channel blockers have no effect; 2. the binding of the compounds do not affect the agonist action of 5-HT at the orthosteric binding site; 3. that once the compounds have bound they completely block conductance through the channel; 4. that where the binding sites of two compounds overlap, only one or other is able to bind at any one time and that the compounds effectively compete for that binding site according to the usual Gaddum competitive model (Gaddum, 1937; Colquhoun, 2006); 5. where the binding sites of two compounds do not overlap, that both compounds are capable of binding independently to their respective sites; 6. binding of the compounds is in equilibrium. In the case of condition 5 it is possible that the binding of one compound may prevent access of another to its own site. However, in practice this will not influence the analysis since the functional consequence of the binding of both compounds compared to one will be the same, namely complete channel blockade. Different sub-IC_50_ concentrations of each of the compounds (BB 16 μM, GB 11 μM and PXN 5 μM) were chosen to allow distinction between the different scenarios.

### 2.6 Radioligand binding

Competition binding (8 point) was performed on at least three separate plates of transfected cells. Briefly, 50 μg of cell membranes were incubated in 0.5 ml ice-cold HEPES buffer (10 mM, pH 7.4) containing 1 nM [^3^H]granisetron in the presence or absence of BB (up to 2.5 mM), GB (up to 2.5 mM) or PXN (up to 5 mM); non-specific binding was determined using 1 mM quipazine. Reactions were incubated for at least 1 h at 4 °C and terminated by vacuum filtration using a Brandel cell harvester onto GF/B filters pre-soaked in 0.3% polyethyleneimine. Radioactivity was determined by scintillation counting using a Beckman BCLS6500 (Fullerton, California, USA). Data were analysed by iterative curve fitting (GraphPad Prism v3.02) according to the equation:

(5) $$B_{L}=B_{\min}+\frac{B_{\max}-B_{\min}}{1+10^{n_{\text{H}}\left(\log\;L_{50}-\log\;L\right)}}$$

where *L* is the concentration of ligand present; *B_L_* is the binding in the presence of ligand concentration *L*; *B*_min_ is the binding when *L* = 0; *B*_max_ is the binding when *L* = ∞, *L*_50_ is the concentration of *L* which gives a binding equal to (*B*_max_ + *B*_min_)/2; and *n*_H_ is the Hill coefficient. Results are presented as mean ± S.E.M.. Statistical analysis was performed using ANOVA in conjunction with a Dunnett’s post-hoc test.

## 3 Results

### 3.1 Inhibition of 5-HT_3_ receptor currents

**Fig. 2:**
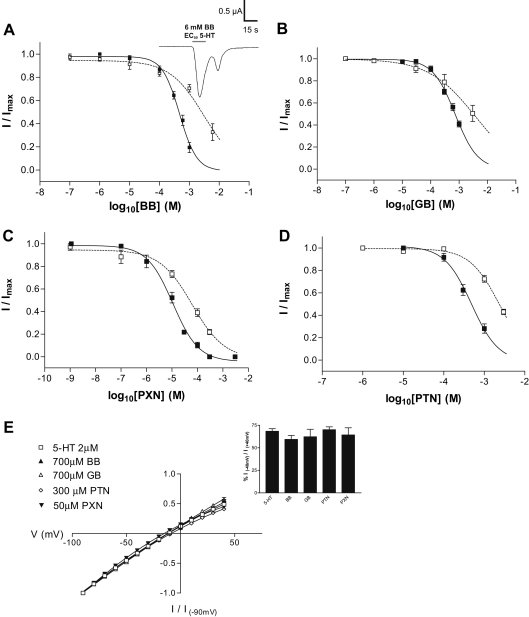
BB (panel A), GB (panel B), PXN (panel C) and PTN (panel D) inhibition of the 5-HT_3_A (filled squares, solid line) and 5-HT_3_AB (open squares, dotted line) receptor *EC*_50_ responses. A *inset* shows the current rebound sometimes observed following the removal of high concentrations of BB. Panel E shows current-voltage relationships for 5-HT_3_A receptor responses in the presence of BB, GB, PXN or PTN. The curves have been normalised to the current at measured at −90 mV, and the i*nset* shows the ratios of current amplitudes recorded at +40 mV relative to those at −40 mV. The ratios in the absence of antagonist were unaltered in the presence of BB, GB, PTN or PXN. GA inhibition is not shown as only minimal inhibition was seen at 1 mM in the 5-HT3A receptor only.

Oocytes injected with cRNA encoding human 5-HT_3_A and 5-HT_3_AB receptors responded to application of 5-HT in a concentration-dependent manner. Data was fitted with Eq. (1) to yield the p*EC*_50_ and Hill Slope values that are shown in Table 1. These are similar to previously published results (Thompson and Lummis, 2008). None of the compounds had effects on uninjected oocytes and none of the compounds elicited a response when applied alone. BB, GA, GB, PTN and PXN caused concentration-dependent inhibition of 5-HT *EC*_50_ responses that was unchanged by pre-application of the compounds (Fig. 2, Table 2). Upon washout of a very high concentration of BB (6 mM), a transient rebound current was observed (channel block is relieved and a brief current flows before the channel closes; Fig. 2A, *inset*), but none of the other compounds showed this behaviour across the range of concentrations shown. The potencies of the compounds were lower at 5-HT_3_AB receptors (Fig. 2, Table 2). As GB and PXN were considerably more potent than GA and PTN, no further work was performed on the latter compounds.

### 3.2 Competition binding

At 5-HT_3_A receptors the p*K*_d_ of [^3^H]granisetron was 9.03 ± 0.06 (*n* = 8, *K*_d_ = 0.93 nM), and at 5-HT_3_AB receptors was 9.04 ± 0.05 (*n* = 6, *K*_d_ = 0.91 nM). Binding of [^3^H]granisetron (1 nM) to human 5-HT_3_A or 5-HT_3_AB receptors was not affected by BB (2.5 mM), GB (2.5 mM) or PXN (5 mM).

PTX-induced inhibition of murine 5-HT_3_ receptor currents has been previously reported, but no radioligand binding data was shown (Das and Dillon, 2003). Here, competition binding with granisetron (0.5 nM) on the mouse 5-HT_3_A (*p*K_d_ = 9.30 ± 0.05, *n* = 11) and 5-HT_3_AB (*p*K_d_ = 9.26 ± 0.20, *n* = 3) receptors showed that this ligand was not displaced by high concentrations of BB (2.5 mM), GB (2.5 mM) or PXN (5 mM).

### 3.3 Mechanism of action

**Fig. 3:**
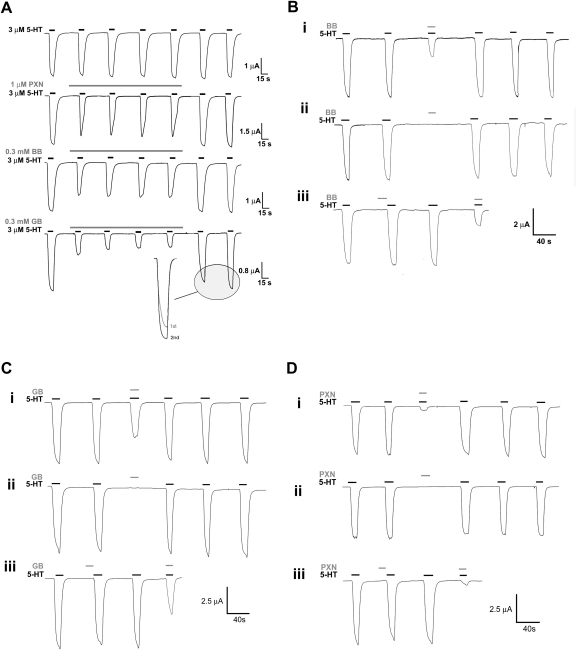
Evidence for channel block. A: The use-dependency observed for GB was absent for BB and PXN. For GB, the percentage inhibition measure at peak (31 ± 3.1%) was less than that measured during the first application (38 ± 0.8%). For GB, full recovery was not seen until a second application of GB was applied (*inset*); at 0.3 mM GB, the first 5-HT response was 91 ± 2% of the second 5-HT response. B–D: The inhibition observed following the co-application (i) of each compound was not seen if the same compound was pre-applied before 5-HT (ii and iii). When compounds were pre-applied immediately before 5-HT (iii), no reduction in the 5-HT response was noted. 5-HT = 2 μM. BB = 1 mM, GB = 300 μM, PXN = 30 μM. For each compound, traces are representative of >5 experiments on ≥2 batches of oocytes.

As the *IC*_50_ values of BB, GB and PXN were high at 5-HT_3_AB receptors, the mode of action was only studied at 5-HT_3_A receptors. Application of each of the compounds in the absence of 5-HT (Fig. 3) did not change the peak response of subsequent 5-HT applications, suggesting that the compounds can only access the binding site in the open state. This suggests open channel block, but examination of inhibition at a ranged of holding potentials revealed that none of the compounds displayed voltage-dependence (Fig. 2D). Use-dependence of BB, GB and PXN was also examined, and Fig. 3A illustrates the currents induced by 3 μM 5-HT applied at 1 min intervals, in the continued presence of antagonist. With BB and PXN, the level of inhibition of the 3 μM 5-HT response remained stable during successive applications. In contrast, the continued presence of 300 μM GB produced a progressive decrease in the peak current with subsequent 5-HT applications, indicating that this compound was use-dependent. When saline rather than GB was perfused between the co-applications this was not seen, suggesting that the behaviour was a consequence of the accumulated inhibition. The GB response only recovered to its pre-treatment amplitude after two agonist applications, showing that recovery was dependent on channel activity (Fig. 3A). Across the range of GB concentrations shown in Fig. 2B, responses did not show increased levels of inhibition with prolonged applications of the compound, and did not rebound following its removal.

**Fig. 4:**
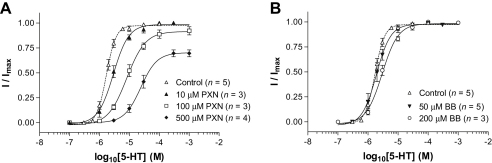
Concentration–response curves in the absence and presence of increasing concentrations of BB and PXN. Increasing the concentration of PXN (Panel A), caused a rightward shift of the concentration-response curves and a change in maximal current that was consistent with non-competitive antagonism. Because of the low potency and poor compound solubility at high concentrations, it was not possible to perform experiments at higher concentrations and observe shifts for BB (Panel B) or GB (data not shown). Because of these limitations, inhibition of the 5-HT_3_ receptor response was recorded at single compound concentrations, both alone and in combination (*see*Table 3).

Concentration-response curves in the presence of increasing concentrations of PXN (between 1–50 × *IC*_50_) displayed rightwards shifts and a reduction in the maximal current (Fig. 4A). The same effects have been previously observed for non-competitive antagonists at 5-HT_3_ receptors (Thompson et al., 2007), and have a variety of possible explanations (see discussion). The low potency of BB and GB meant we had insufficient quantities to perform experiments across a similar range of compound concentrations. To overcome this limitation we developed the mathematical model that is presented below.

### 3.4 Co-application

To probe independence of binding sites at the 5-HT_3_A receptor, we compared the levels of inhibition displayed by two compounds applied separately or together (Table 3). Predicted values for the level of inhibition expected with both compounds were derived as described in Material and Methods. For each combination of BB, GB and PXN, the observed level of inhibition was closest to the predicted value using Scenario 1 (common binding regions). For BB /GB and BB /PXN, the observed data was not significantly different from the predicted values using Scenario 1, but was significantly different from those using Scenario 2. These data suggest that these compounds share at least part of the same binding site. For GB /PXN, the differences between the observed and predicted levels of inhibition were not significant.

## 4 Discussion

The ginkgolides are a class of therapeutic agents that can act at a variety of anion-selective Cys-loop receptors. Here we show that they can also act at cation selective receptors: BB and GB inhibit 5-HT_3_A receptor function by blocking the receptor channel, although they are ∼100 fold less potent than at GABA and glycine receptors (Huang et al., 2004; Hawthorne et al., 2006), suggesting that it is not action at this excitatory Cys loop receptor that contributes to their anxiolytic – or other *in vivo* – properties. Nevertheless these compounds are potentially useful pharmacological tools as they can discriminate between homomeric (5-HT_3_A) and heteromeric (5-HT_3_AB) receptors, a property shared by PTX, but few other compounds. We also show that PXN, which is structurally related to the ginkgolides, is the main active component of PTX at the 5-HT_3_A receptor and has a site of action that overlaps with that of BB.

PTX has previously been shown to inhibit 5-HT_3_ receptors by blocking the channel (Das and Dillon, 2003, 2005). As our data indicated GB and BB do not act at the orthosteric binding site, we predicted that these compounds would also block the channel, and, if so, may bind at the same location as PTX. To test this hypothesis we set up a simple mathematical model to analyse the results of co-application experiments. The question we were seeking to address was whether the extent of blockade of an open channel was the same when two compounds acting in the channel compete for a single site or bind independently to separate sites. In either case, the binding of one single compound would cause complete functional blockade. A statistical analysis of the data revealed that in the 5-HT_3_A receptor the BB binding site overlaps with that of GB and PXN (Table 3), supporting our hypothesis that these three compounds block the 5-HT_3_A receptor channel by binding in a similar location. The same analysis was not performed on 5-HT_3_AB receptors which required much higher concentrations for inhibition.

Most of the work on ginkgolide action at Cys loop receptors has been performed on GABA and glycine receptors, where BB, GB and PTX at low micromolar concentrations have been shown to bind to and block the receptor channel: In the GABA_A_ receptor, for example, PTX protects against MTS modification of residues at the cytoplasmic end of M2, and mutations in this region effect PTX inhibition, but not GABA *EC*_50_ or benzodiazepine modulatory effects (Sedelnikova et al., 2006; Ticku et al., 1978; Xu et al., 1995). *In silico* docking into GABA_A_ and glycine receptors shows that ginkgolides and PTX can dock in the pore, and has provided plausible binding locations and ligand orientations (Hawthorne and Lynch, 2005; Erkkila et al., 2008; Hawthorne et al., 2006; Jensen et al., 2008; Yang et al., 2007). Our data show that the 5-HT_3_A receptor channel is also the most likely site of action as BB and GB do not displace the competitive antagonist [^3^H]granisetron, the effects of PXN on 5-HT-evoked currents were insurmountable, and there is overlap between the BB and PXN, and BB and GB binding sites.

For PXN a reduction in the maximal current and a rightward shift of the concentration–response curve was seen. True non-competitive antagonists have a constant *IC*_50_ regardless of the agonist concentration, although some open channel blockers can cause an apparent increase in affinity as the antagonist effectively becomes less active at low agonist concentrations, because there are fewer receptors in the activated state. Apparent decreases in affinity (i.e. rightwards shifts), however, have been observed for some channel blockers, and mixed competitive /non-competitive behaviours or receptor reserve are often used to explain these data (e.g. Buisson and Bertrand, 1998). Concentration-response curves could be similarly altered if the antagonist preferentially binds to a specific receptor state, or there are different on and off rates of the agonist /antagonists concerned (see e.g. Smart and Constanti, 1986). Thus our data have various possible explanations, but do not prove or disprove open channel block. Voltage-dependence is also often cited as evidence for open channel block, but, as all the compounds that displayed inhibitory behaviours in this study were uncharged, it is not surprising that their actions were voltage-independent. To further probe channel block we examined use dependence. Our experiments show a weak use-dependence with GB supporting open channel block, and, although this was not seen for BB and PXN, recovery from GB inhibition was dependent on channel activity, which is also characteristic of open channel block. We conclude that these compounds occupy the open channel, but may access the binding site slowly and /or preferentially bind to a closed state.

This study also demonstrates that PXN is the active component of PTX at 5-HT_3_ receptors. PTX is a well established channel blocker at a range of Cys-loop receptors, and at the majority of receptors, PXN is more potent than PTN, e.g. in the GABA_A_ receptor PXN is ∼50 fold more potent than PTN, and *∼*30 fold more potent in glycine α2 receptors (Chen et al., 2006; Curtis and Johnston, 1974; Yang et al., 2007). However, there are exceptions as PXN and PTN have similar *IC*_50_ values at glycine α1 (∼5 μM) and α1β (∼30 μM) receptors (Yang et al., 2007). Structurally, PTN is almost identical to PXN (only differing in hydroxylation of the isoprenyl side chain; Fig. 1), indicating that the protein-receptor interaction at the 5-HT_3_ receptor is highly specific. Similarly, ginkgolides, which have very subtle chemical differences, can have different relative potencies at different Cys-loop receptors suggesting they have specific binding sites; the differing behaviours we observed at the 5-HT_3_A receptor with GA and GB also indicate a specific interaction (Chatterjee et al., 2003; Heads et al., 2008; Huang et al., 2004 Jaracz et al., 2004).

The current study also demonstrates that BB, GB and PXN have distinct potencies at 5-HT_3_A and 5-HT_3_AB receptors, with *IC*_50_ values ∼ 6 fold higher at heteromers. This is consistent with previous work on PTX that also showed a 6-fold potency difference between these receptor types (IC_50_s derived from data in Holbrook et al., 2009, using *n*_H_ = 1 and plateau = 100), and supports our hypothesis that all these compounds have similar sites of action. In another study Das and Dillon (2005) using murine receptors reported an ∼40 fold difference in the *IC*_50_ values of PTX (5-HT_3_A, 41 μM; 5-HT_3_AB, 1.1 mM), and also showed that an M2 residue (6′) was a common determinant of PTX inhibition. Thus, while it is possible that the difference in potency is due to different behaviours at these two subtypes, as, for example, has been described for methadone (Deeb et al., 2009), we speculate that the mechanisms of action of the structurally-related gingkolide compounds are similar in homomeric and heteromeric receptors, and it is the different pore lining residues that are the cause of the different potencies.

In conclusion, we have shown that BB and GB are inhibitors of 5-HT_3_ receptors, and that PXN is the active component of PTX. At 5-HT_3_A and 5-HT_3_AB receptors the ligands have different potencies, extending the range of compounds that can differentiate between homo- and heteromeric 5-HT_3_ receptor types. The data show that the sites of action for BB, GB and PXN in 5-HT_3_A receptors are in the receptor channel, and their sites overlap. Our results are consistent with the behaviours of these compounds at other Cys-loop receptors, demonstrating that their mechanism of action is broadly conserved across the family.

## Figures and Tables

**Table 1:** Parameters obtained from concentration–response curves.

Receptor	p*EC*_50_ (M)	*EC*_50_ (μM)	Hill slope	*n*
5-HT_3_A	5.76 ± 0.03	1.73	2.56 ± 0.31	6
5-HT_3_AB	4.55 ± 0.05	28.1	1.00 ± 0.12	8

Values are mean ± SEM.

**Table 2:** Parameters obtained from concentration-inhibition curves.

Compound	p*IC*_50_ (M)	*IC*_50_ (μM)	Hill slope	*n*
*5-HT_3_A*				
BB	3.33 ± 0.03	470	1.48 ± 0.16	6
GA	<3.0	>1000	–	4
GB	3.14 ± 0.05	730	1.10 ± 0.13	4
PTN	3.33 ± 0.05	470	1.33 ± 0.15	6
PXN	4.97 ± 0.12	11	0.68 ± 0.12	13

*5-HT_3_AB*				
BB	2.50 ± 0.10	3200	0.75 ± 0.13	4
GA	<3.0	>1000	–	4
GB	2.41 ± 0.22	3900	0.52 ± 0.14	4
PTN	2.53 ± 0.04	2900	0.99 ± 0.07	4
PXN	4.20 ± 0.11	62	0.68 ± 0.10	5

Values (mean ± SEM) were derived from concentration–inhibition curves of wild type 5-HT_3_ receptors expressed in *Xenopus* oocytes. Inhibition was recorded at the 5-HT EC_50_ concentration at both 5-HT_3_A (1.7 μM) and 5-HT_3_AB (28 μM) receptors. Concentration–inhibition curves for GA did not converge as inhibition did not exceed 50% at the highest concentration used (1 mM).

**Table 3:** Determination of shared or discrete binding sites.

Level of inhibition (1 − (*I*/*I*_max)_)				Scenario 1 prediction	Scenario 2 prediction	ANOVA *p*	*n*
BB	GB	PXN	Combined				
0.30 ± 0.06	–	0.48 ± 0.04	0.54 ± 0.03*	0.57 ± 0.04*^†^	0.63 ± 0.05^†^	0.049	5
0.13 ± 0.01	0.25 ± 0.01		0.33 ± 0.01*	0.33 ± 0.02*	0.35 ± 0.02^†^	0.012	4
	0.26 ± 0.05	0.35 ± 0.03	0.47 ± 0.04	0.46 ± 0.04	0.50 ± 0.05	0.629	7

BB, GB, PXN and Combined are experimentally derived. The concentrations of BB (16 μM), GB (11 μM) and PXN (5 μM) were sub-IC_50_ levels chosen for easy comparison. Scenarios 1 and 2 are the predicted levels of inhibition for scenarios 1 (shared binding site) and 2 (discrete binding sites), as described in Materials and Methods. For each pairing, ANOVA was performed to determine the significance of differences between Combination, Scenario 1 and Scenario 2. When the result of ANOVA was significant, homogeneous subsets are identified by * and ^†^.
